# A proteomic approach to identifying spermatozoa proteins in Indonesian native Madura bulls

**DOI:** 10.3389/fvets.2023.1287676

**Published:** 2023-12-04

**Authors:** Zulfi Nur Amrina Rosyada, Berlin Pandapotan Pardede, Ekayanti Mulyawati Kaiin, Muhammad Gunawan, Tulus Maulana, Syahruddin Said, Ligaya I. T. A Tumbelaka, Dedy Duryadi Solihin, Mokhamad Fakhrul Ulum, Bambang Purwantara

**Affiliations:** ^1^Division of Reproduction and Obstetrics, School of Veterinary Medicine and Biomedical Sciences, IPB University, Bogor, Indonesia; ^2^Research Center for Applied Zoology, National Research and Innovation Agency (BRIN), Bogor, Indonesia; ^3^Division of Veterinary Anatomy, Faculty of Veterinary Medicine, Universitas Airlangga, Surabaya, Indonesia; ^4^Department of Biology, Faculty of Science, IPB University, Bogor, Indonesia

**Keywords:** fertility, food security, LC–MS/MS, Madura bulls, sperm proteins fertility, proteomic, sperm proteins

## Abstract

Proteins assist sperm mature, transit the female reproductive tract, and recognise sperm oocytes. Indigenous Indonesian bulls, Madura bulls, have not been studied for reproductive proteomics. As local Indonesian beef livestock, Madura cattle assist in achieving food security; hence, their number must be improved. Thus, the identification of molecular proteomics-based bull fertility biomarkers is needed. This study aimed to characterise the sperm fertility function of the superior Madura bull (*Bos indicus* × *Bos Javanicus*) spermatozoa proteome. Frozen semen from eight Madura superior bulls (*Bos indicus* × *Bos javanicus*) aged 4–8 years was obtained from the artificial insemination centre (AIC) in Singosari and Lembang. Madura superior bulls are those that have passed the bull breeding soundness evaluation. Frozen sperm were thawed and centrifuged at 3000 × g for 30 min. Proteins in sperm were characterised through proteomic analysis using liquid chromatography–tandem mass spectrometry (LC–MS/MS). The resulting gene symbols for each protein were then subjected to bioinformatics tools, including UniProt, DAVID, and STRING databases. Regarding sperm fertility, the analysis revealed that 15 proteins were identified in the sperm of Madura bulls. Amongst the identified proteins, the superior Madura bull sperm contained several motilities, energy-related proteins, and chaperone proteins. A substantial portion of characterised proteins are linked to metabolic pathways and the tricarboxylic acid (TCA) cycle, contributing to sperm energy production. In conclusion, the first in-depth proteome identification of sperm related to sperm quality and bull fertility of a unique indigenous Madura breed of Indonesia was performed using the LC–MS/MS proteomic method. These findings may serve as a reference point for further studies related to the functions of bovine sperm and biomarkers of fertility and sperm quality.

## 1 Introduction

Recent developments in proteomics have significantly impacted our understanding of how sperm become fertile (1). One of the most differentiated cells is the sperm head, which has a highly compacted chromatin structure and an enormous midpiece that contains the machinery required to interact and transfer paternal genetic and epigenetic information to the oocyte (2). Due to their high level of differentiation, sperm is suitable for studying proteomic sections, such as the sperm membrane, which is the most crucial part because of its role in interacting with the environment and the oocyte (3). The fusion of a sperm and an oocyte requires complex membrane modifications of the sperm (4). Spermatozoa proteins that regulate normal/abnormal sperm function have been identified through proteomic investigation (5).

Thus, bull fertility is linked to many proteins involved in spermatogenesis. The significance of specific proteins in controlling sperm quality and fertilization is unknown, although their availability and quantity may alter sperm fertility (6). According to Peddinti et al. (1), many bovine fertility protein markers have been reported. Major semen proteins, such as binding sperm protein (BSP) in Frisian Holstein cattle (6), zona pellucida binding protein (ZPBP) in Bali Polled bulls (7), A-kinase anchoring protein 3 in buffalo (8), and osteopontin in Limousin cattle (9), have been widely reported. The sperm proteome profile of mammals has been studied, including pigs (10), equines (11), sheep (12), and bovines (1). Nevertheless, the sperm proteome remains unknown in Madura bulls.

Madura cattle (*Bos indicus* × *Bos javanicus*) are native to Madura, Indonesia. Small-scale producers raise Madura cattle as working cattle to maintain the regional culture, which still values Sonok and Karapan cattle (13). In addition, Madura cattle are currently being developed as beef cattle using artificial insemination and reproduction technology. It also ensures food security for the Indonesian population. In particular, these cattle are highly valued for their unique reproductive traits, with regular calving even under low-input regimes and in dry and arid regions (14). However, it remains unknown whether the proteome level of Madura bull sperm affects sperm function and fertility. Proteomics offers various methods. However, complex protein analysis using LC–MS has become the preferred analytical technique for quantitative proteomics (15, 16).

Ultimately, acquiring knowledge about the proteome of spermatozoa can provide a holistic understanding of reproductive processes, not only specific to the Madura bull breed but also applicable to bovine species. This study aims to provide a comprehensive profiling of the sperm proteins associated with fertility function in the superior Madura bull (*Bos indicus* × *Bos javanicus*) spermatozoa proteome. Additionally, we used the literature to explain how Madura bull sperm proteins affect bull fertility. Further, proteomic approaches have made it possible to identify proteins that have the potential to function as indicators of male fertility, as well as proteins that are involved in the functional characteristics of sperm.

## 2 Materials and methods

### 2.1 Frozen semen samples

A total of 8 superior Madura bulls (*Bos indicus* × *Bos javanicus*) aged 4 to 8 years with spermatozoa motility >70%, based on secondary data from each AIC in Lembang and Singosari, Indonesia, were included in this study. Frozen semen from each of the eight Madura superior bulls was obtained from the National AIC in Singosari and Lembang Bank of Semen. Madura superior bulls are those that have passed the bull breeding soundness evaluation. The Animal Care and Use Committee excluded this study from its ethical review because artificial vaginal semen collection did not alter the physiology of animals. The study was directed by veterinarians from both institutions and followed SNI ISO 9001: 2015 No. 824 100 16072 at Lembang AIC and SNI ISO 9001: 2015 No. G.01-ID0139-VIII-2019 at Singosari AIC. The ethics committees of Lembang AIC and Singosari AIC provided ethical guidance and sanctions for the responsible collection of bull sperm. Additionally, an experienced bull technician collected sperm using an artificial vagina.

Semen samples from Lembang AIC were processed according to the AIC’s standard operating procedure (SOP), which used skim milk as the extender. The formulation of the skimmed milk diluent in a volume of 1000cc consisted of 100g of skimmed milk, 960cc of distilled water, and an antibiotic solution containing 3000000 IU of penicillin, 3g of streptomycin, and 30cc of distilled water. The ratio of skimmed milk diluent to antibiotic was 100:1. In contrast, the semen samples obtained from Singosari were cryopreserved utilising tris egg yolk as a cryoprotectant. The tris egg yolk extender was formulated using the following components: 20% egg yolk, 1.6% tris aminomethane, 1.4% lactose, 2.5% raffinose, 0.9% citric acid, and an antibiotic mixture consisting of 1000000 IU/L of penicillin, 1g/L of streptomycin, and 1g/L of distilled water. The AIC in Lembang and Singosari employ extenders consisting of skim milk and tris egg yolk supplemented with antibiotics. The extender utilised in every artificial insemination facility serves as a cryoprotectant, safeguarding sperm cells during the freezing process.

Additionally, it enhances semen volume, sustains sperm viability, and regulates sperm pH. Consequently, the inclusion of an extender is imperative in the manufacture of frozen semen to ensure the preservation of sperm quality. The cryopreserved sperm samples were transferred with a specialised transport container that maintained a temperature of −196°C through liquid nitrogen. The pieces were placed within the container for subsequent examination.

### 2.2 Sperm protein isolation

**Figure 1:**
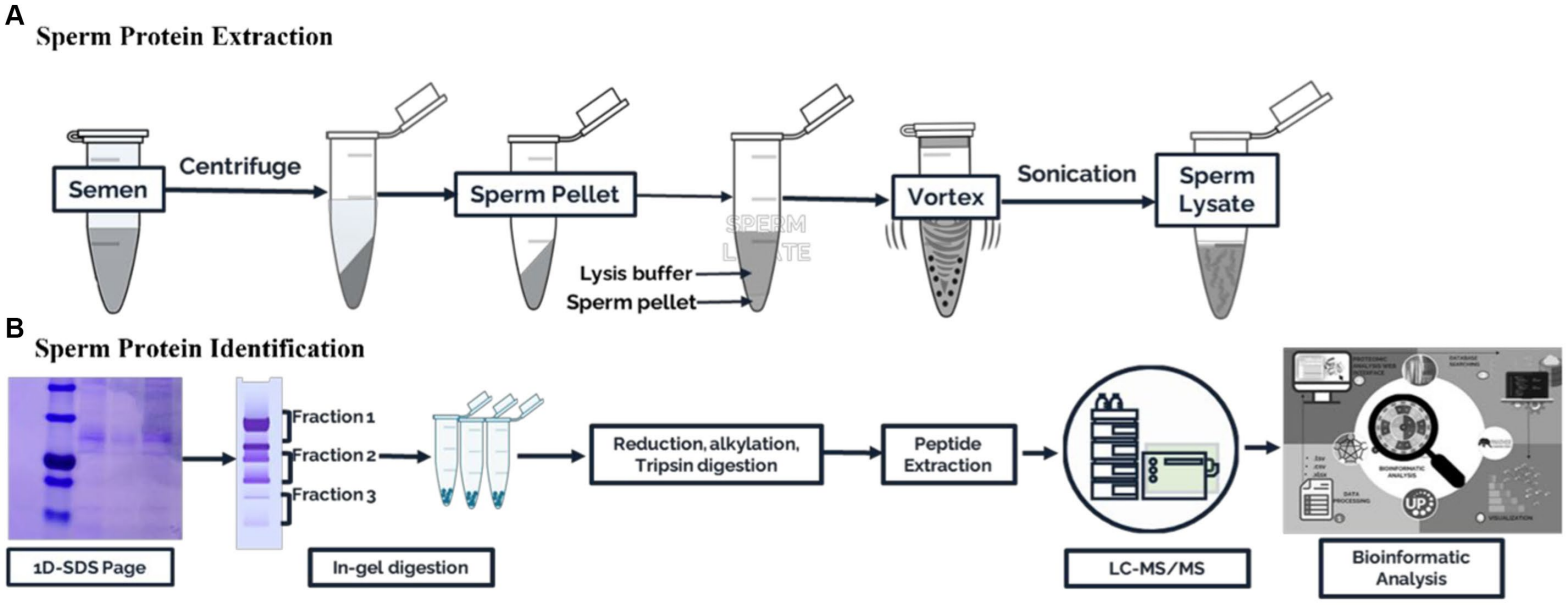
Schematic workflow for the proteomic analysis of Madura bull spermatozoa. (**A**) Sperm protein extraction. **(B)** Sperm protein identification.

The collected semen samples of about ive straws of frozen semen from each of the eight bulls were washed in 2 mL of phosphate buffer saline and centrifuged twice (3000 × g for 10 min, 4°C) to clean and separate the spermatozoa, seminal plasma, and extender of frozen semen. The resulting sperm cell pellet was then resuspended in cell lysis buffer (2% SDS in 62.5 mM Tris–HCl, pH 6.8, 1.0 mM phenylmethanesulfonyl fluoride, and 23 mM benzidine as a protease inhibitor) and stored at −20°C for protein extraction. The sperm pellet was vortexed for 10 min before protein extraction. Next, sonication was used thrice for 20 s each to dissolve the protein. After centrifuging the cell lysates (10,000 × g, 10 min), protein lysates were isolated. The Bradford method (17) was used to estimate the total protein yield of the sperm protein lysate. The Bradford protocol was performed using the Coomassie protein kit instructions (Merck, Darmstadt, Germany). The data were processed using ThermoScan RE software version 3.2 Multiskan Go (Thermo Fisher Scientific, Waltham, Massachusetts, United States). Pools of sperm protein containing equal amounts from three samples were frozen at −20°C for later use. A summary of the experimental design is shown in Figure 1.

### 2.3 Fractionation by one-dimensional sodium dodecyl sulfate-polyacrylamide gel electrophoresis (1D-SDS–PAGE) and in-gel digestion

Following the separation of 35 μg of sperm protein (an equivalent quantity of protein amalgamated from three cases) by 12.5% sodium dodecyl sulfate-polyacrylamide gel electrophoresis (SDS–PAGE), the gel was stained with colloidal Coomassie brilliant blue R-250 (CAS number: 6104-59-2) from HiMedia. Subsequently, the gel bands were excised and digested using a previously described protocol (18). The proteins were reduced using 0.5 M dithiothreitol (DTT) at 55°C for 25 min, followed by alkylation with 14 mM iodoacetamide (IAA) for 40 min at room temperature without light. Gel fragments were treated with trypsin (Promega, Madison, Fitchburg, WI, USA) at a ratio of 1:50 (trypsin: protein) at 4°C for 30 min. The gel fragments were incubated for 18 h at 37°C in a ThermoMixer shaker incubator block (Eppendorf). The reaction in each vial was terminated by adding 1% trifluoroacetic acid (TFA). An extraction buffer (2% TFA in 20% acetonitrile) was used to extract peptide digests from the gel pieces, followed by 70% acetonitrile. The obtained peptide digests were subjected to a drying process and subsequently purified using a C18 spin column for desalination (Thermo Scientific, Pierce Biotechnology, N Meridian Rd., Rockford, IL, USA) and then preserved at −20°C until LC–MS/MS analysis.

### 2.4 Liquid chromatography–tandem mass spectrometry (LC–MS/MS) analysis

The peptides obtained from each sample were subjected to LC–MS/MS analysis using an Ultimate 3000 Nano LC system coupled with a Q-Exactive Plus Orbitrap HRMS system (Thermo Fisher Scientific, Bremen, Germany). The peptides were introduced onto a preanalytical column with dimensions of 75 μm ID, 15 cm length, and 100 pore size, packed with Acclaim PepMap C18 2 μm particles. The solvent used for loading was solvent A, consisting of 0.1% formic acid, and the flow rate was set at 300 nL/min. A Q-Exactive Plus Orbitrap mass analyser was used for subsequent analysis. The peptides were subsequently resolved on an analytical column (50 cm 75 m ID, Pep Map RSLC C18 2 m) at a flow rate of 300 nL/min for 120 min using an increasing gradient of 5–35% solvent B (98% acetonitrile and 0.1% formic acid). Peptide signals were collected using an LTQ-Orbitrap mass spectrometer (Thermo Fisher Scientific, Bremen, Germany). The MS spectra within the 200–2000 m/z range were obtained using an Orbitrap analyser with a resolution of 30,000 (at m/z 400). Subsequently, ten precursor MS scans were conducted using collision-induced dissociation fragmentation at 35% normalised collision energy (19).

### 2.5 Protein database searching and bioinformatics analysis

Protein analysis was performed on proteins that contained at least one unique peptide. The exclusion of specific peptides based on the “minimum two-peptide rule may result in the perpetual disregard of genuine peptides, and proteins must have a sequence score HT > 0. Thus, we manually confirmed single peptide spectral match (PSM)-identified protein MS/MS spectra. Protein data were acquired and analysed using the UniProt bovine protein database (http://www.uniprot.org) and Proteome Discover version 2.2 software (Thermo Fisher Scientific). Furthermore, the discovered proteins from spermatozoa were also put into functional groups based on Gene Ontology (GO) using the DAVID web resource (https://david.ncifcrf.gov/tools.jsp). The interactions between the proteins were obtained using STRING version 11.0 (https://string-db.org/).

## 3 Result

### 3.1 Protein characterisation of Madura bull spermatozoa

**Figure 2:**
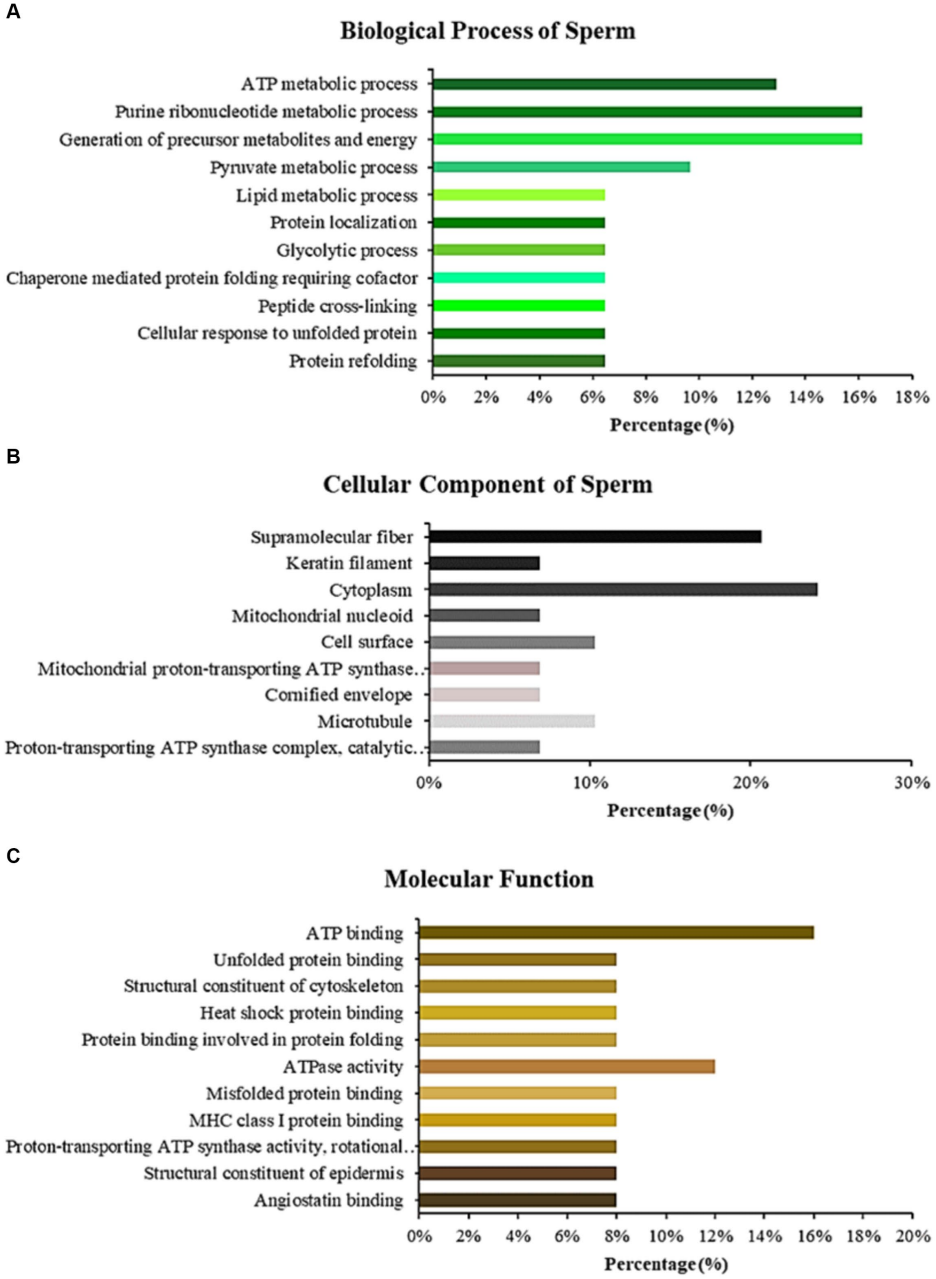
Bar chart representing the gene ontology annotations of proteins identified in Madura bull spermatozoa according to biological processes **(A)**, cellular components **(B)**, and molecular functions **(C)**.

LC–MS/MS identified Madura bull sperm protein markers of fertility and environmental compatibility. All the databases contained in the database were only *Bos taurus* origin proteins since *Bos indicus* × *Bos javanicus* proteins are limited. This study analysis identified 15 proteins in Madura bull (*Bos indicus* × *Bos Javanicus*) sperm using UniProt and DAVID software (Table 1). Fifteen proteins were identified that were associated with sperm function categories (Table 1), including adenosine triphosphate (ATP) synthesis activity, cellular metabolic processes, cilia/flagella, sperm motility, capacitation and acrosome reaction, sperm-egg fusion, spermatogenesis and fertilization, and chaperone proteins. Functional annotation of proteins from Madura bull spermatozoa was further carried out using DAVID software and divided into several categories: “Biological Process” (BP), “Cellular Component” (CC), and “Molecular Function” (MF) (Figure 2). Although different categories were created for each division, the most numerous were those involved in the generation of precursor metabolites and energy (16%), urine ribonucleotide metabolic processes (16%), ATP metabolic processes (13%), and pyruvate metabolic processes (10%) in the case of BP (Figure 2A); cytoplasm (24%), supramolecular fibre (21%), and cell surface and microtubule (10%) in CC (Figure 2B); and ATP-binding proteins (16%), ATPase activity (12%) and unfolded protein binding (8%) in MF (Figure 2C).

**Table 1:** Proteins identified in Madura bull sperm associated with sperm function.

Protein accession	Gene symbol	Protein name	Function	Molecular weight (kDa)
A0A4 W2HIH5	AKAP4	A-kinase anchoring protein 4	flagellated sperm motility, motile cilium assembly	99.6
A0A4 W2BT96	ATP5F1A	ATP synthase F1 subunit alpha	lipid metabolic process, ATP synthase activity	63.1
A0A452DII8	ATP5F1B	ATP synthase F1 subunit beta	ATP synthesis activity, Hydrogen ion transport, Ion transport	62.2
A0A6P5BW43	ACO2	aconitase 2	tricarboxylic acid cycle, citrate metabolic process	85.3
A0A4 W2CUL5	COL4A2	collagen type IV alpha 2 chain	cell membrane receptors, metabolic processes, spermatozoa differentiation,	179.5
A0A4 W2EVB5	C1orf56	Chromosome 1 open reading frame 56	Capacitated sperm and acrosome reaction	37.9
A0A3Q1M0V5	ENO3	Enolase 3	glycolytic process, pyruvate metabolism, ATP formation from ADP	62.1
A0A4 W2CEF3	GAPDHS	glyceraldehyde-3-phosphate dehydrogenase, spermatogenic	glucose metabolism, glycolytic process, formation of ATP from ADP, spermatozoa energy precursor	44.1
A0A4 W2D4U4	HSPA2	heat shock protein family A (Hsp70) member 2	male meiosis I, spermatogenesis, spermatid development, response to heat and cold stress, positive regulation of G2/M transition of the mitotic cell cycle, cell differentiation, positive control over ATPase activity, protein folding mediated by chaperones that require cofactors, the reaction of cells to unfolded proteins, and protein refolding	69.81
A0A4 W2DHF8	HSPA9	heat shock protein family A (Hsp70) member 9	ATP binding, metabolic processes, mitochondrial protein folding, enzyme binding, stress response	73.7
A0A4 W2I3C7	SPAM1	sperm adhesion molecule 1 (Hyaluronidase)	Sperm egg recognition, fertilization	62.4
A0A6P5CLI1	ODF2	the outer dense fiber of sperm tails 2	spermatogenesis, cell differentiation, cilium organisation, regulation of cilium assembly,	105
A0A6P5BWX7	PDHA2	pyruvate dehydrogenase E1 subunit alpha 2	Carbohydrate metabolism, Glucose metabolism,	43.3
Q3MHM5	TUBB4B	tubulin beta 4B class IVb	microtubule-based process, Cytoskeleton	49.8
F6RP72	TUBB1A	Tubulin alpha chain	microtubule-based process, Cytoskeleton	50.9

### 3.2 Protein interaction networks and pathway enrichment analysis

**Figure 3:**
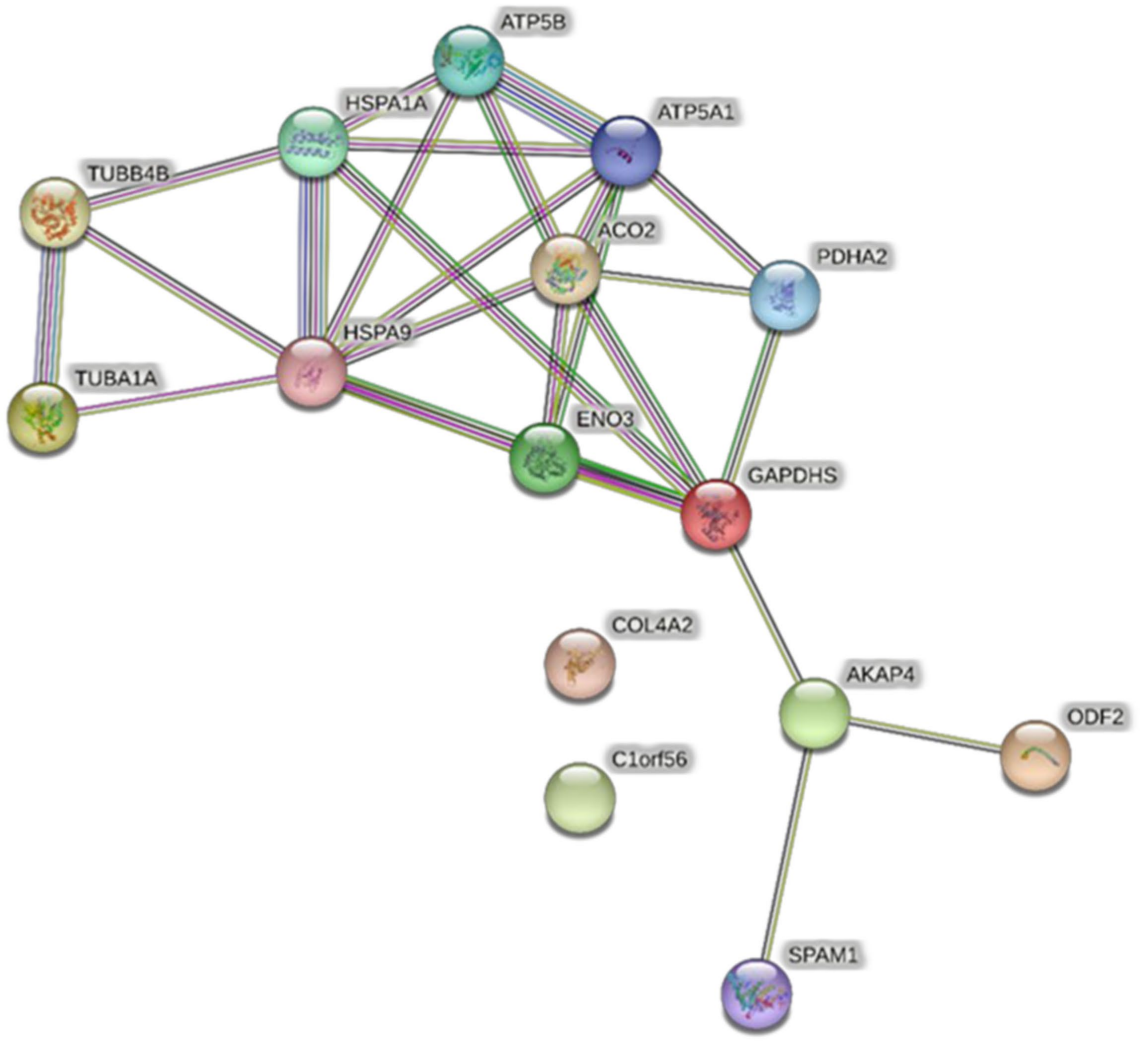
STRING protein–protein interaction network showing the interactions of the spermatozoa proteins identified in Madura bull sperm.

**Figure 4:**
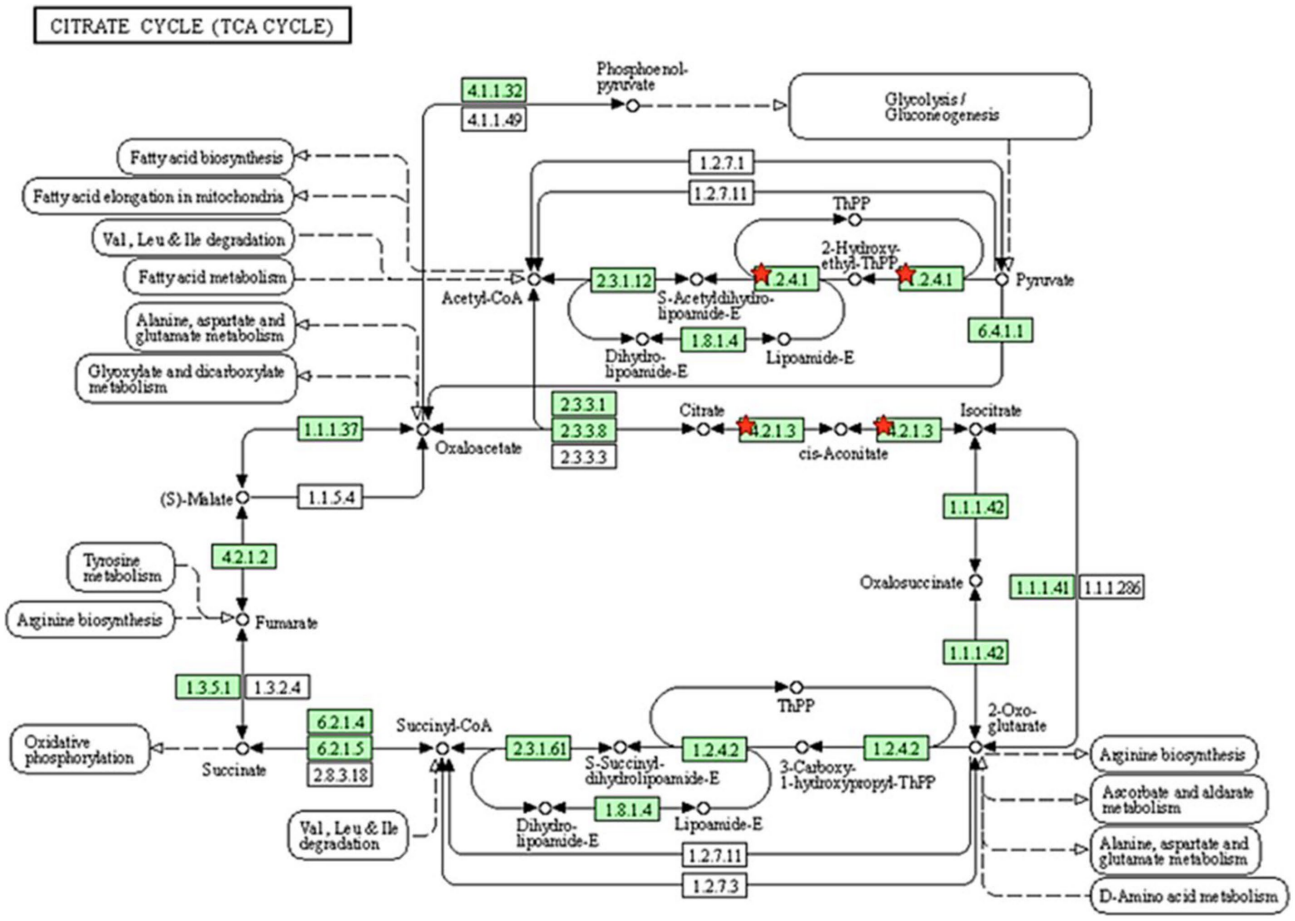
Pathway analysis using the KEGG pathway database identified proteins involved in the TCA cycle. The red star denotes the detected proteins.

**Figure 5:**
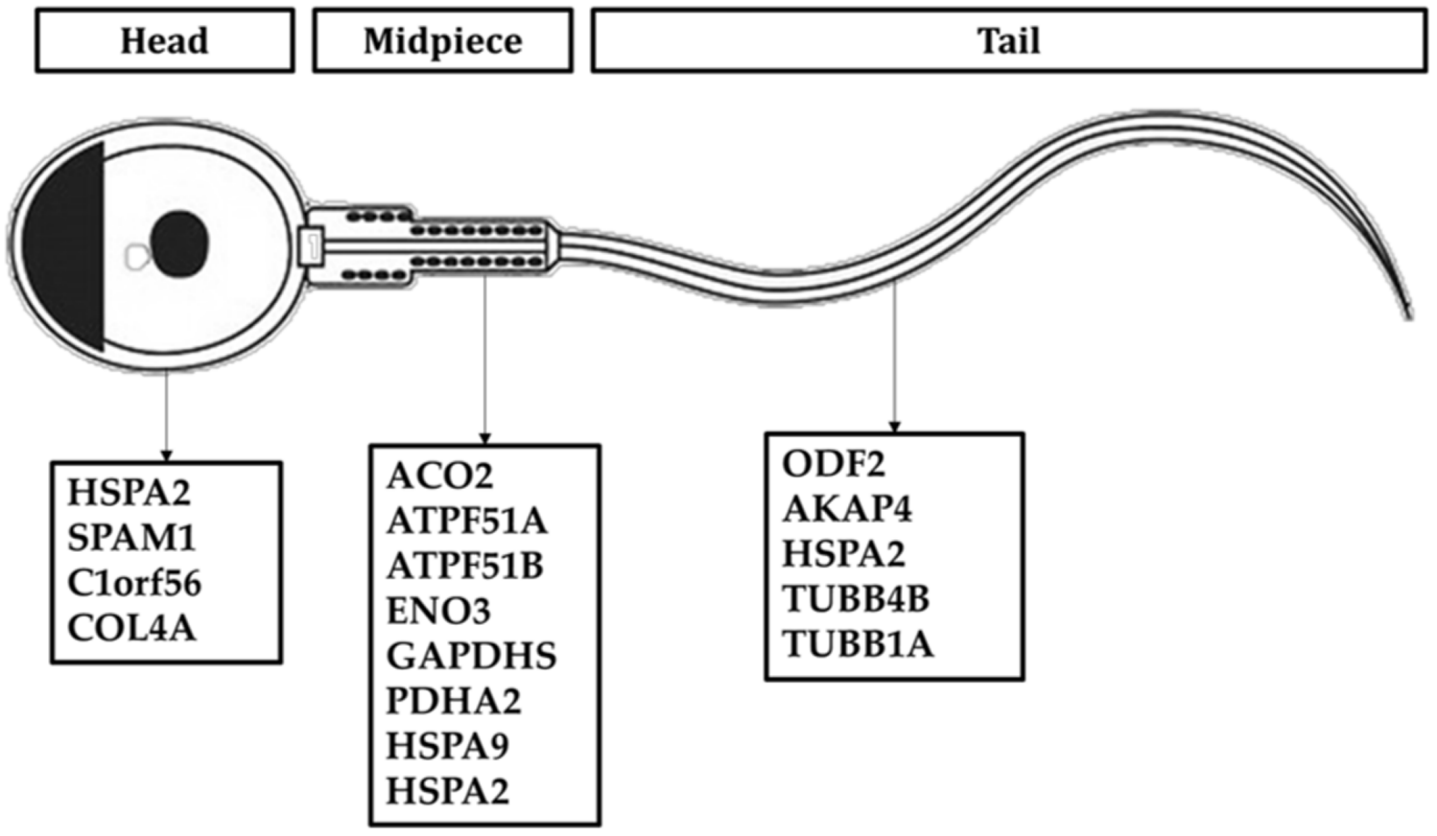
Distribution of sperm protein of Madura bull (*Bos indicus* × *Bos javanicus*).

All proteins identified in Madura bull spermatozoa were then searched using STRING software (version 11.0) for protein–protein interaction network analysis. Light blue lines show protein nodes, whereas pink lines reflect experimentally established interactions. The expected interactions are green for the gene neighbourhood, red for gene fusion, and dark blue for gene co-occurrence. Other protein relationships are shown by light green text mining, black co-expression, and blue protein homologies. A coloured node shows the query protein, and a white node leads the second shell of the interaction. Proteins with unknown 3D conformations had empty nodes, whereas those with known conformations had full nodes. The interactions between the 15 identified proteins are shown in Figure 3. Fifteen proteins were directly or indirectly connected through one or more interacting proteins, indicating functional links. We categorised proteins using the Kyoto Encyclopaedia of Genes and Genomes (KEGG) pathway terminology to study Madura bull sperm pathways. As expected, many of the discovered proteins were involved in metabolic pathways. The discovered proteins were associated with the TCA cycle (Figure 4). The sperm head, mitochondria (mid-piece), and flagellum/tail expressed the majority of these proteins (Figure 5).

## 4 Discussion

Fifteen proteins found in the sperm of Madura bulls were found to be related to fertility based on LC–MS/MS analysis (Table 1). The utilisation of known molecular weight standards for calibration is a fundamental aspect of size-exclusion chromatography that provides insights into the distribution of molecular weights. The current study has demonstrated that Madura bull sperm exhibits a molecular weight distribution of proteins within the 40 to 180 kDa range. This study utilised GO annotation to ascertain the cellular localisation, biological processes, and molecular functions of proteins specific to the sperm of Madura bulls.

### 4.1 Protein related to sperm fertility function

Effect of sperm motility on semen quality Male infertility is often attributed to reduced sperm motility. Therefore, the motility proteins of the Madura bull breed must be identified. We found many of these proteins in the spermatozoa. SPAM1 is a transmembrane protein present in ejaculated bull spermatozoa. It originates from the testis and occupies the anterior head region, where the orientation of the C-terminus with the zonal-binding domain facilitates interaction with zona pellucida after the acrosome reaction (20). This protein has also been detected in male sperm (21). The roteomee analysis carried out in this study from the sperm of Madura Bull also revealed the existence of the hyaluronidase protein, also known as SPAM1, isoform 62.4 kDa.

The flagellum of mammalian spermatozoa has intricate supplemental structures around the core axoneme, including the outer dense fibers (ODFs). ODFs protect the sperm tail against shear stress that may arise during epididymal transit and ejaculation. Four major proteins (ODF1, ODF2, ODF3, and ODF4) are amongst more than 14 polypeptides produced by mammalian ODFs. In mice, disrupting ODF2 expression lowers sperm motility, which is consistent with the characteristics of asthenozoospermia (22). Moreover, the cervical midpiece junction requires the presence of ODF2, which comprises part of the centrosome and is separated from the flagellum (23). Our analysis identified ODF2 in Madura bull spermatozoa.

In addition, we found that enolase protein, previously discovered in the plasma membrane of sperm from Bali bulls (24), was present in sperm samples from Madura bulls. During glycolysis and gluconeogenesis, enolase reversibly converts 2-phosphoglycerate into phosphoenolpyruvate. This enzyme produces sperm motility energy (25). Xi et al. (26) found a significant positive correlation between ENO3 and sperm motility parameters in sturgeons. Similarly, He et al. (25) discovered ENO3 in the midpiece and tail of ram sperm, which strongly correlated with sperm motility. The presence of ENO3 expression in Madura cattle sperm and its correlation with sperm function, particularly motility, can be readily explained.

The present study revealed the detectability of AKAP 4 in Madura bull sperm using LC–MS/MS. AKAP4, a marker involved in flagellar structure and motion, is associated with the equine sperm tail (27) and is substantially preserved in mice (28), bulls (29), and humans (30). AKAP4 attaches cAMP-dependent protein kinase A (PKA) to different subcellular locations. In the sperm fibrous sheath, AKAP4 binds PKA and interacts with other proteins to regulate motility. AKAP4, a critical fibrous sheath protein, bundles glycolytic enzymes and phosphorylation-signalling cascade components to supply a localised source of ATP and govern flagellar motion, sperm motility, and hypermotility. Many glycolytic enzymes closely linked to the fibrous sheath at the primary component of the flagellum are required for sperm motility (27).

Tubulins were also found to be another type of protein that contributes to sperm motility that was uncovered in this study. Tubulins are proteins found in the microtubules of sperm (30). Two tubulin proteins were identified as present in the sperm of the studied Madura bull samples. These proteins are known as TUBA1A and TUBB4B. These proteins are necessary for cilia and flagella formation; hence, their importance cannot be overstated.

### 4.2 Chaperone proteins: heat shock proteins

Heat stress affects cattle productivity and reproduction. Heat stress damages developing spermatozoa. However, heat tolerance can be inherited by offspring (3). According to the relevant literature, Madura cattle are a native breed of beef cow thought to have developed on the dry and barren island of Madura (3). Madura Island receives 1600 mm of rain annually and is hot and arid (31). HSPs are linked to heat tolerance and reproductive performance (3, 32). In this study, HSPA9 and HSPA2 were found to be ubiquitously expressed. HSPA9, found in sperm mitochondria, significantly reduces the effects of heat stress in tropical cattle (33, 34). It is also known to regulate sperm motility through metabolic processes, ATP binding, and folding of mitochondrial proteins (35).

HSPA2 (also known as HSP70-2) was discovered in this study, which is consistent with previous findings in Australian Brahman bulls (*Bos indicus*) (36) and Zebu (37). HSP70-2 (heat shock protein 70–2) was found to be expressed in the sperm nucleus as well as in various other sperm organelles, including the mitochondria and flagellum (Figure 5). HSP70-2 is a component of the chromatin structure and contributes to gene regulation, particularly in the folding and unfolding of proteins. This HSPA2 sperm protein may also be crucial in protecting the sperm of tropical acclimated bulls such as Madura from heat or environmental stress. HSP70 is also an excellent predictor of thermotolerance and thermoresistance (38). The potential use of HSP70 as a biomarker for animal fertility and thermotolerance has been suggested (39). Therefore, HSP70-2 is recognised as a dual-functioning gene (3).

### 4.3 Pathway enrichment

The enriched pathways were also analysed for the identified proteins in the Madura bull spermatozoa. The present study revealed that 16% of the proteins were involved in the biosynthesis of precursor metabolites and energy. Only metabolic pathways were included in the list of the significant ways enriched by the proteins. Energy metabolism is essential for sperm development. Sperm require ATP, which is most likely to maintain morphological changes during the spermatid stage, for the degradation and synthesis of active proteins (40). ATP also serves as the molecular motor that provides the necessary energy for flagellar movement in all kinetics-related biochemical activities. The idea that mitochondria in the sperm midpiece drive mammalian sperm is evolving, as evidence suggests that glycolysis is the preferred metabolic pathway to maintain sperm motility in many species (41). Thus, the current study’s results are in line with earlier research showing that ATP synthases are hub proteins found in the sperm of rams that are comparatively less fertile and may regulate how much ATP is used by sperm throughout the fertilization process (42). Existing data indicate that ATP production in sperm is facilitated by both glycolysis and mitochondrial respiration. These processes are interdependent and regulate sperm function based on the availability of energy substrates in the surrounding environment. An alternative glycolysis pathway for ATP production is also found in stallion spermatozoa (43). This study presents novel findings that suggest that Madura sperm are dependent on mitochondrial ATP synthesis, specifically regarding the roles of ATPF51A and ATP5B in sperm function and fertility.

Further, glucose utilisation is restricted during spermatogenesis, and lactate and pyruvate are favoured as substrates for energy synthesis (19). The generation of ATP in mammalian sperm is attributed to the energy metabolism pathway. This pathway exhibits subcellular compartmentalisation, with oxidative phosphorylation (OXPHOS) predominantly occurring in the sperm midpiece and glycolysis in the principal piece (40). Glycolysis is a significant mechanism for facilitating the transport of ATP along the flagellum. Westhoff and Kamp (44), as well as Welch et al. (45), have reported the presence of glyceraldehyde 3-phosphate dehydrogenase (GAPDH) in significant quantities within the fibrous sheath of sperm from different mammalian species, including humans. GAPDH is a NAD-dependent glycolytic enzyme responsible for facilitating the conversion of glyceraldehyde 3-phosphate (GAP) to 1,3-biphosphoglycerate (1,3 BPG). Notably, one of the isoforms of GAPDH, known as GAPDHS, has been identified in this study.

Moreover, Zini et al. (46) found that an augmentation in the activity of pyruvate dehydrogenase complexes can increase pyruvate-lactate usage, thus producing a more significant amount of NADH. NADH oxidase can utilise the NADH produced to generate reactive oxygen species (ROS) necessary for the capacitation process. NADH oxidase activity and ROS formation have also been associated with the motility of human spermatozoa. In this way, GAPDHS contributes significantly to energy metabolism. Elkina et al. (47) found GAPDHS in the testes of humans and rats; the enzyme is primarily present in the cytoplasm of all spermatogenic cells, whereas it is localised in the sperm tail in the epididymis. This finding provides support for this theory. This previous study by Kumar et al. (48) also established the involvement of extramitochondrial localised pyruvate dehydrogenase complex (PDHA) in the process of sperm capacitation, as well as the significance of pyruvate in the overall energy metabolism of mammalian sperm. The metabolic pathway encompassed the GAPDHS and PDHA2 proteins, both detected in the sperm of Madura bulls in the present study.

In agreement, energy metabolism plays a critical role in facilitating sperm function. The TCA cycle is the primary energy source for spermatids, although glycolytic and pentose phosphate pathways also play a role in energy synthesis in spermatozoa. The TCA cycle generates adenine, which is converted to ATP by the electron transport chain of the OXPHOS pathway. These proteins may be involved in creating the acrosome and activities there, both of which require energy provided by oxidative phosphorylation. ATP is then delivered to the microtubules responsible for supporting various sperm functions (49). In this study, two candidate proteins related to the tricarboxylic acid cycle pathway were PDHA2 and ACO2.

It was intriguing to discover that specific proteins are involved in so many energy metabolism processes, suggesting that a single protein may not perform glycolysis or OXPHOS as the primary actor in maintaining sperm functionality. The current study shows that proteomic methods may provide a reliable source for sperm protein detection and improve gene ontology comprehension. Therefore, we used high-throughput LC–MS/MS to develop a proteomic profile of Madura bull sperm connected to reproductive characteristics. Preserving superior native germplasm and preventing gene pool depletion requires studying protein differences in different breeds. Thus, investigating Madura bull protein composition can help us comprehend native Indonesian bull sperm biology. Finally, knowing the proteome of ejaculated spermatozoa can help us understand the reproductive process in Madura bull and bovine in general.

## 5 Conclusion

We examined the sperm proteomes of Madura cows (*Bos indicus* × *Bos javanicus*). The use of several methods to prepare samples has made it easier to identify proteins in sperm. Most of the proteins identified in this study are essential for bull breeding. We believe that information about the sperm proteome of the superior Madura bull, a breed with good reproductive traits, will speed up future studies on bull fertility. Additionally, this would enable the creation of molecular tools for accurately selecting bulls to conserve indigenous Indonesian cattle breeds and bovines.

## Data availability statement

The original contributions presented in the study are included in the article/supplementary material, further inquiries can be directed to the corresponding authors.

## Ethics statement

Ethical approval was not required for the studies on animals in accordance with the local legislation and institutional requirements because only commercially available established cell lines were used.

## Author contributions

ZR: Conceptualization, Data curation, Writing – original draft, Writing – review & editing, Formal analysis, Investigation, Methodology. BP: Data curation, Funding acquisition, Project administration, Validation, Writing – original draft, Writing – review & editing, Formal analysis, Investigation, Methodology, Visualization. EK: Writing – review & editing. MG: Writing – review & editing. TM: Writing – review & editing. LT: Writing – review & editing, Conceptualization, Supervision. DS: Conceptualization, Supervision, Writing – review & editing. MU: Conceptualization, Supervision, Writing – review & editing. BP: Conceptualization, Data curation, Funding acquisition, Project administration, Supervision, Validation, Writing – original draft, Writing – review & editing. SS: Methodology, Supervision, Formal analysis, Validation, Investigation, Funding acquisition, Writing – review & editing.
